# Apocynin attenuates left ventricular remodeling in diabetic rabbits

**DOI:** 10.18632/oncotarget.16599

**Published:** 2017-03-27

**Authors:** Jiuchun Qiu, Jianping Zhao, Jian Li, Xue Liang, Yajuan Yang, Zhiwei Zhang, Xiaowei Zhang, Huaying Fu, Panagiotis Korantzopoulos, Gary Tse, Tong Liu, Guangping Li

**Affiliations:** ^1^ Tianjin Key Laboratory of Ionic-Molecular Function of Cardiovascular Disease, Department of Cardiology, Tianjin Institute of Cardiology, Second Hospital of Tianjin Medical University, Tianjin, People's Republic of China; ^2^ First Department of Cardiology, University of Ioannina Medical School, Ioannina, Greece; ^3^ Department of Medicine and Therapeutics, Chinese University of Hong Kong, Hong Kong, SAR, P.R. China; ^4^ Li Ka Shing Institute of Health Sciences, Chinese University of Hong Kong, Hong Kong, SAR, P.R. China

**Keywords:** left ventricular remodeling, diabetes mellitus, oxidative stress, apocynin, NADPH oxidase

## Abstract

**Introduction:**

Nicotinamide adenine dinucleotide phosphate (NADPH) oxidases are responsible for the generation of reactive oxygen species, producing vascular and myocardial dysfunction in diabetes mellitus. However, the potential benefits of the NADPH oxidase inhibitor, apocynin, on left ventricular (LV) remodeling remain unknown.

**Results:**

In the diabetic group, interventricular septal thickness and left ventricular posterior wall thickness were markedly increased compared to control. These changes were accompanied by increased LV cardiomyocyte cross-sectional area and greater degree of interstitial fibrosis. NO, myeloperoxidase, and malonaldehyde levels in the serum were significantly increased Moreover, protein expression levels of rac1, nuclear factor-κB, transforming growth factor-β, p38, P-p38, and metalloproteinase-9 were also raised. Apocynin treatment prevented all of these structural, histological and biochemical changes and additionally increased superoxide dismutase levels.

**Methods:**

Thirty Japanese rabbits were randomized into three groups: control, alloxan-induced diabetes with and without apocynin treatment at 15 mg/kg/day for 8 weeks (*n* = 10 for each group). Echocardiography was performed and hemodynamics were assessed by carotid and LV catheterization. LV cardiomyocyte cross-sectional area and interstitial fibrosis were evaluated by histology. Serum nitric oxide (NO), malonaldehyde, myeloperoxidase, superoxide dismutase (SOD) levels, and activity of LV tissue NADPH oxidases was assessed. Expression of proteins involved in pro-inflammatory and pro-fibrotic signaling were determined by Western blotting.

**Conclusions:**

Inhibition of NADPH oxidase using apocynin is an effective upstream therapy for preventing diabetes-induced adverse remodeling of the left ventricular myocardium.

## INTRODUCTION

The prevalence of type 2 diabetes mellitus is expected to reach 5.4%, affecting approximately 300 million by 2025 globally [1]. Diabetes, especially if glycemic control is poor, causes damage to multiple organ systems, leading to significant morbidity and mortality. For example, it can result in cardiac hypertrophy, cardiomyopathy and heart failure [2, 3]. Histologically, this is reflected by the increased amount of fibrotic tissue within the myocardium [4]. In addition to mechanical dysfunction, this can lead to impaired conduction of action potentials [5], increasing the likelihood of re-entrant arrhythmias and sudden cardiac death [6].

Recent research efforts have focused on elucidating the molecular mechanisms by which increased oxidative stress is generated in diabetes, and how this leads to vascular and cardiac dysfunction [7–9]. Recently, our group demonstrated that higher levels of oxidative stress were associated with increased atrial fibrosis in an alloxan-induced diabetic rabbit model [10–13]. In the ventricles, reactive oxidant species (ROS) can also promote cardiac hypertrophy, fibrosis, contractile dysfunction and eventually pump failure [14]. The primary sources of ROS include the nicotinamide adenine dinucleotide phosphate (NADPH) oxidases, mitochondria, xanthine oxidases, and uncoupled NO synthases (NOS) [14], while NADPH oxidase (NOX) activates redox signaling, in turn causing tissue injury [15, 16].

Apocynin is an effective inhibitor of NADPH oxidase [17] by preventing p47phox assembly to the enzyme core [18]. In this study, we tested the hypothesis that this agent may protect against ventricular hypertrophy and fibrosis in an alloxan-induced diabetes model in rabbits. This was achieved by experiments conducted at different levels of complexity, from hemodynamic and mechanical functions at the whole organ level, to determination of protein levels and activity of pro- and anti-oxidant enzymes at the tissue level.

## RESULTS

### Body weight, blood, hemodynamic and echocardiographic parameters

Our initial experiments confirmed that alloxan successfully induced diabetes in the rabbits after 6 weeks, leading to lower body weight, increased glucose levels and decreased insulin levels compared to control (Table 1) (*P* < 0.05). Apocynin treatment did not alter these parameters. There were no differences in blood urea nitrogen, creatinine, total cholesterol, triglyceride or low density lipoprotein levels between the three groups. Whilst high density lipoprotein was not significantly different between the control and diabetic groups, we observed a reduction in its levels with the apocynin group.

**Table 1:** Body weight and blood parameters

	Control group (*n* = 10)	Diabetes group (*n* = 10)	Apocynin group (*n* = 10)
Weight, kg	2.71 ± 0.28	2.32 ± 0.36*	2.53 ± 0.49
Glucose, mmol/L	5.43 ± 0.75	17.72 ± 6.19*	19.61 ± 5.21
Insulin, uIU/mL	16.04 ± 1.40	7.56 ± 1.34*	7.01 ± 1.21
Blood Urea Nitrogen (mmol/L)	6.54 ± 1.26	7.14 ± 1.36	7.28 ± 1.01
Creatinine (umol/L)	91.60 ± 12.18	99.74 ± 10.80	104.50 ± 12.05
Total cholesterol (mmol/L)	1.63 ± 0.13	1.84 ± 0.28	1.50 ± 0.53
Triglyceride (mmol/L)	1.13 ± 0.34	1.39 ± 0.47	1.26 ± 0.52
High Density Lipoprotein (mmol/L)	0.94 ± 0.28	0.90 ± 0.30	0.58 ± 0.20#
Low Density Lipoprotein (mmol/L)	0.59 ± 0.10	0.70 ± 0.29	0.61 ± 0.14

Values are mean ± SD; *Compared with control (*P* < 0.05). #Compared with the diabetic group (*P* < 0.05).

**Figure 1:**
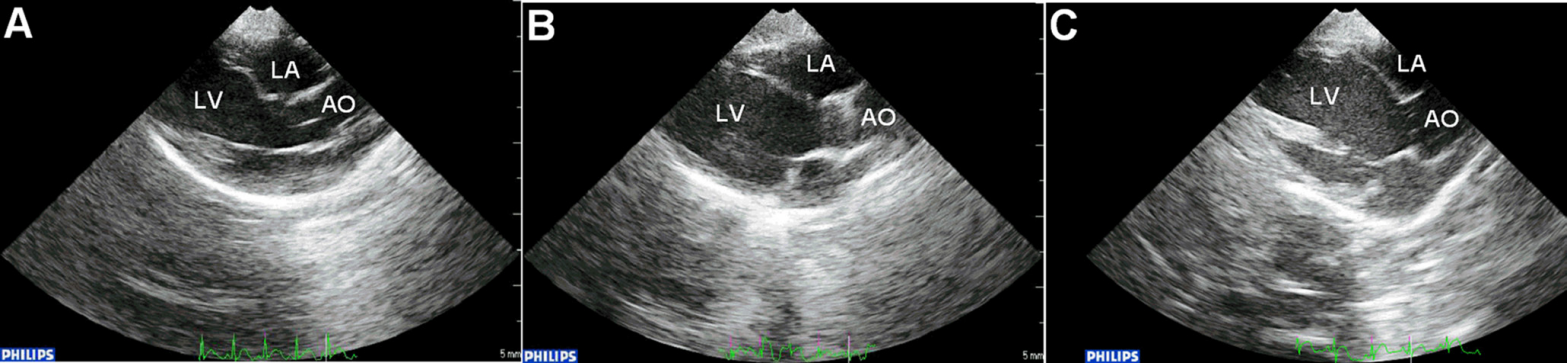
Representative parasternal long-axis echocardiographic views in the control (**A**), diabetic group (**B**) and apocynin treatment group (**C**). Abbreviations: LA = left atrium; LV = left ventricle; AO = aorta.

No significant differences in systolic and diastolic blood pressures, or in left ventricular end-point pressure were observed between controls, diabetes group alone and diabetes group with apocynin treatment (Table 2). Representative echocardiographic images in the parasternal long axis view are shown in Figure 1. Left atrial antero-posterior diameter, interventricular septal thickness and left ventricular posterior wall thickness were significantly increased in the diabetes group (*P* < 0.05). Increases in the latter two parameters were attenuated by apocynin treatment (*P* < 0.05). No differences in left ventricular end-diastolic or end-systolic diameters or in left ventricular ejection fraction were observed (*P >* 0.05).

**Table 2:** Hemodynamic and echocardiographic parameters

	Control group(*n* = 10)	Diabetes group(*n* = 10)	Apocynin group(*n* = 10)
Systolic blood pressure, mmHg	112.5 ± 7.53	113.7 ± 6.90	115.4 ± 8.81
Diastolic blood pressure, mmHg	85.2 ± 5.49	86.6 ± 5.34	90.3 ± 8.21
Left ventricular end-diastolic pressure, mmHg	−7.4 ± 2.32	−6.0 ± 5.31	−8.6 ± 2.80
Left atrial antero-posterior diameter, mm	6.88 ± 0.42	8.53 ± 0.81*	7.77 ± 1.25
Interventricular septal thickness, mm	1.71 ± 0.09	2.13 ± 0.25*	1.91 ± 0.14#
Left ventricular posterior wall thickness, mm	1.67 ± 0.07	2.10 ± 0.25*	1.89 ± 0.13#
Left ventricular end-diastolic diameter, mm	12.4 ± 0.64	13.42 ± 1.44	13.60 ± 1.43
Left ventricular end-systolic diameter, mm	7.12 ± 0.57	7.90 ± 1.39	7.72 ± 0.50
Left ventricular ejection fraction, %	72.14 ± 6.0	73.55 ± 9.41	74.88 ± 6.70

Values are mean ± SD; *Compared with control (*P* < 0.05). #Compared with the diabetic group (*P* < 0.05).

### Left ventricular hypertrophy and interstitial fibrosis

**Figure 2:**
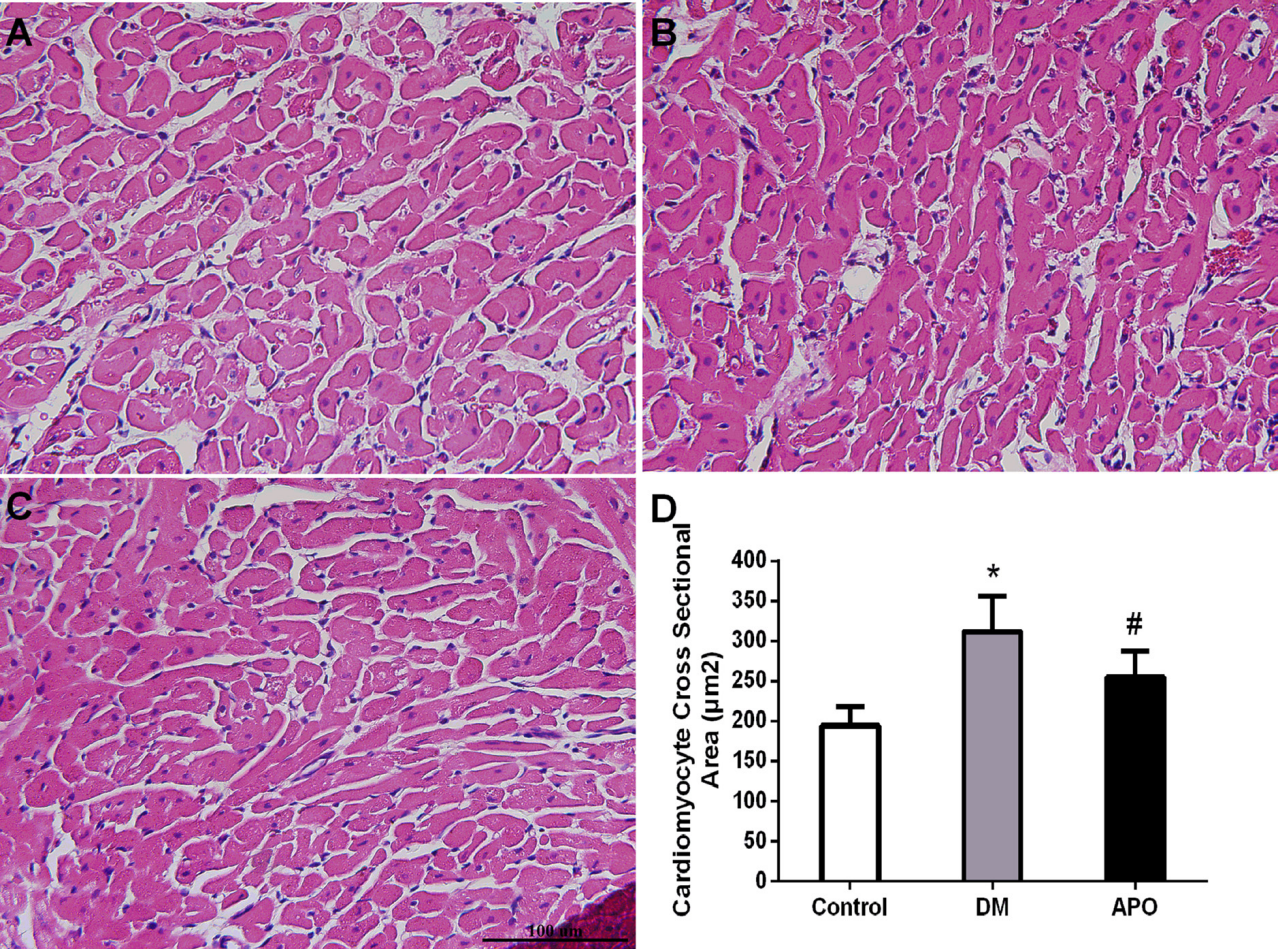
Left ventricular cardiomyocytes (×400) evaluated with H&E staining in the control (**A**), diabetic group (**B**) and apocynin treatment group (**C**). (**D**): Demonstrates cardiomyocyte mean cross sectional area in each groups. * and # indicate significant difference when apocynin treatment group was compared with control group and the diabetes group, respectively (*P* < 0.05).

**Figure 3:**
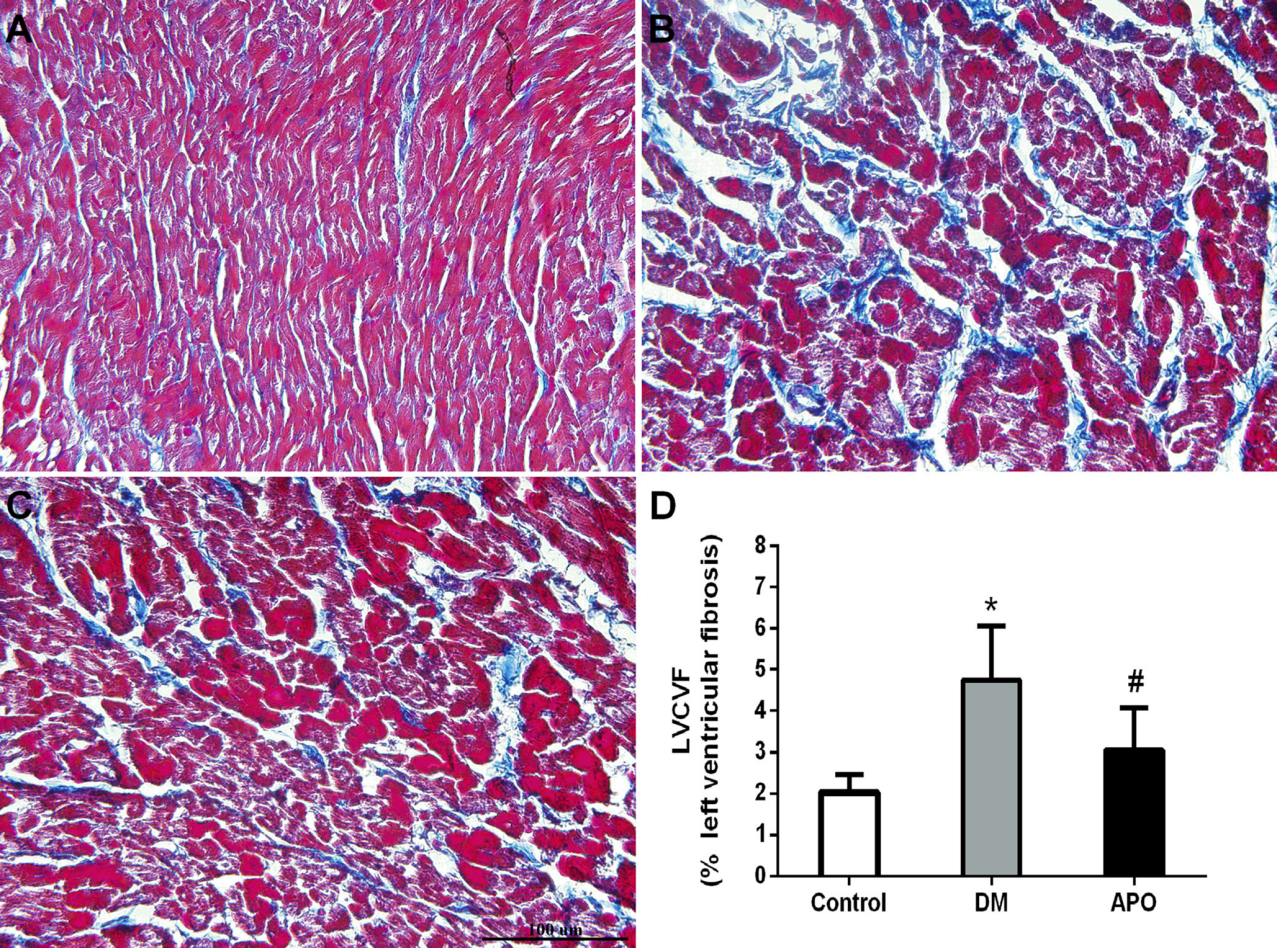
Interstitial fibrosis (×400) in the control (**A**), diabetic group (**B**) and apocynin treatment group (**C**). (**D**): Left ventricular interstitial collagen volume fraction (LVCVF), expressed as a percentage in each group. * and # indicate significant difference when apocynin treatment group was compared with control group and the diabetes group, respectively (*P* < 0.05).

After echocardiographic and hemodynamic assessments, the hearts were weighed as a whole and for the left ventricle only. Whole heart weight and left ventricular weight ratios were significantly increased in the diabetic group compared with controls but not further altered by apocynin (*P* < 0.05) (Table 3). Mean cross-sectional area of cardiomyocytes from the left ventricle was higher (Figure 2) and greater amount of interstitial fibrosis, as measured by the left ventricular interstitial collagen volume fraction (Figure 3), were observed in the diabetes group compared to control (*P* < 0.05). These changes were prevented by apocynin treatment (*P* < 0.05).

**Table 3:** Heart weights, oxidative stress parameters and NADPH oxidase activity

	Control group(*n*=10)	Diabetes group(*n*=10)	Apocynin group(*n*=10)
Heart weight ratio, 1/1,000	2.24 ± 0.11	2.41 ± 0.16*	2.32 ± 0.21
LV weight ratio, 1/1,000	1.53 ± 0.07	1.75 ± 0.14*	1.66 ± 0.14
Nitric oxide, umol/L	94.99 ± 14.24	137.08 ± 25.43*	109.02 ± 22.17#
Superoxide dismutase,U/ml	458.22 ± 63.46	484.80 ± 67.29	565.10 ± 70.76#
Myeloperoxidase, U/L	51.23 ± 9.40	60.18 ± 8.56*	53.20 ± 6.91
Malonaldehyde, nmol/ml	9.70 ± 2.27	14.47 ± 2.74*	10.88 ± 1.70#
NADPH oxidase activity,umol NADPH/min/mg protein	22.69 ± 6.06	49.79 ± 8.78*	33.18 ± 8.30#

Values are mean ± SD; *Compared with control (*P* < 0.05). #Compared with the diabetic group (*P* < 0.05). NADPH = nicotinamide adenine dinucleotide phosphate.

### Oxidative stress parameters and western blotting for protein expression levels

Table 3 summarizes the oxidative stress parameters. Compared with the control group, serum nitric oxide, myeloperoxidase and malondialdehyde levels, as well as NADPH oxidase activity were significantly increased in the diabetic group (*P* < 0.05). Apocynin significantly decreased serum nitric oxide and malondialdehyde levels and NADPH oxidase activity back to control values (*P* < 0.05) and increased serum SOD levels (*P* < 0.05).

**Figure 4:**
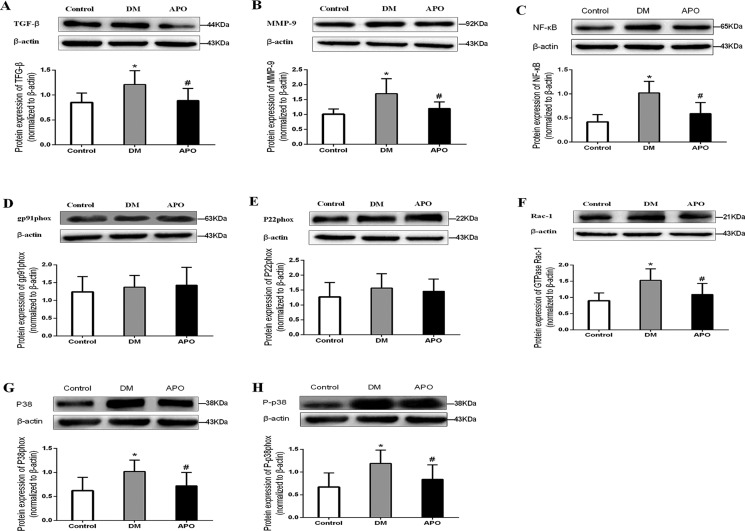
Western blotting results for transforming growth factor-beta (TGF- β, **A**), metalloproteinase-9 (MMP-9, **B**), nuclear factor kappa B (NF-κB, **C**), gp91phox (**D**), p22phox (**E**), Ras-related C3 botulinum toxin substrate 1 (rac1, **F**), p38 (**G**), phosphorylated p38 (**H**) normalized to β-actin. Values are expressed as mean ± SD * and # indicate significant difference when apocynin treatment group was compared with control group and the diabetes group, respectively (*P* < 0.05).

Figure 4 shows representative Western blots for the protein expression involved in pro-inflammatory and fibrotic signaling pathways. Expression levels of transforming growth factor-beta (TGF-β, A), metalloproteinase-9 (MMP-9, B), nuclear factor kappa B (NF-κB, C), Ras-related C3 botulinum toxin substrate 1 (rac1, F), p38 (G) and phosphorylated p38 (H) were significantly in the diabetic group compared to controls. All of these changes were prevented by apocynin treatment. By contrast, expression of gp91phox (D) and p22phox (E) were not significantly different between the three groups (*P* < 0.05).

## DISCUSSION

The major findings of the present study are as follows: Diabetes (1) induced left ventricular hypertrophy without altering mechanical function as evidenced by echocardiography, (2) increased mean cross-sectional area of cardiomyocytes and increased interstitial fibrosis, (3) increased serum nitric oxide, myeloperoxidase and malondialdehyde levels, (4) increased NADPH oxidase (NOX) activity and (5) increased protein expression levels of several pro-inflammatory and pro-fibrotic signaling components, including transforming growth factor-beta (TGF-β), metalloproteinase-9 (MMP-9), nuclear factor kappa B (NF-κB), Ras-related C3 botulinum toxin substrate 1 (rac1), p38 and phosphorylated p38. (6) The NOX inhibitor, apocynin, prevented most of these pathological changes, and additionally increased superoxide dismutase levels.

Higher levels of oxidative stress are known to promote inflammation, fibrosis, and apoptosis [19]. Cellular hypertrophy and fibrosis, features commonly observed in diabetic cardiomyopathy [4], are promoted by increased reactive oxygen species (ROS) production [20–23]. In diabetic cardiomyopathy, interstitial fibrosis occurs primarily as a result of increased type III collagen deposition [22–24]. Hyperglycemia enhances the glycation of proteins and lipids [25], and activates NOX via protein kinase C (PKC) [26]. NOX is a major source of ROS [14] and plays a critical role in hyperglycemia-induced oxidative stress in the heart [27]. Increased NOX-dependent ROS production has been confirmed in hearts isolated from diabetic animals and cultured cardiomyocytes exposed to high glucose concentrations [28, 29]. The catalytic subunits of NOX include gp91phox and p22phox. Our Western blot experiments showed no differences in p22phox and gp91phox between control, diabetes alone or diabetes treated with apocynin. Rac1 is a cofactor for assembly of the active NOX complex [30, 31]. It has been shown that cardiomyocyte-specific Rac1 deficiency reduced ROS production and prevented cardiac fibrosis and hypertrophy in STZ-induced diabetic mice [32]. In our experiments, we demonstrated upregulation of Rac1 in diabetes. These findings suggest that enzyme activation rather than transcriptional control was responsible for the enhanced NOX activity [33, 34].

Increased oxidative stress can activate NF-κB, a major transcriptional regulator that can induce the expression of TGF-β, resulting in fibrosis [35]. The mitogen-activated protein kinase family is involved in a diverse number of regulatory processes, include response to growth signals, apoptosis, and stress [15]. One of these kinases is the p38 MAPK. In isolated perfused rat hearts, p38-MAPK are activated by increased oxidative stress [36]. In our study, the fact that expression of this kinase was also raised suggested that cardiac fibrosis and hypertrophy may be dependent on this signaling pathway.

There are many approaches that can be used to reduce oxidative stress. Oxidation processes of proteins and lipids by ROS may be so rapid, that scavenging antioxidants, such as vitamins C and E, may not able to counteract their actions. Indeed, experimental evidence suggests that upstream therapies may be more effective to prevent oxidative damage by inhibiting ROS formation [37], rather than acting to reduce their damage once they are formed. A potential target would be NOX, which is inhibited by the agent apocynin. Previous experiments have shown that apocynin can prevent endothelial dysfunction in diabetic rats [38]. It ameliorated ventricular hypertrophy and fibrosis in a rabbit myocardial infarction model of heart failure [18]. It also decreased tachycardia-induced TGF-β1-mediated atrial fibrosis [39].

In conclusion, our data demonstrate that apocynin can prevent structural remodeling in diabetes. Further studies are needed to examine whether it can similar prevent abnormal electrophysiological remodeling to reduce the incidence of ventricular arrhythmias in diabetic cardiomyopathy.

## MATERIALS AND METHODS

### Study design and preparation of the rabbit DM model

The experiments described in this study was approved by the Experimental Animal Administration Committee of Tianjin Medical University. Thirty Japanese rabbits (1.8–2.5 kg) were obtained from Beijing Medical Animals Research Institute (Beijing, China). They were randomly assigned into 3 groups (*n* = 10 for each group): control, alloxan-induced diabetic group, and diabetic group treated with oral apocynin (Sigma, Saint Louis, MO, USA) at 15mg/day/kg.

In the diabetes groups, alloxan monohydrate (Sigma, Saint Louis, MO, USA) was dissolved in sterile normal saline to achieve a concentration of 5% (W/V), and a dose of 120 mg/kg was quickly administered intravenously into the marginal ear vein. The presence of diabetes mellitus was confirmed 48 hours later by blood glucose levels showing values ≥ 14 mmol/L (one measurement) or ≥ 11 mmol/L (two measurements). Blood glucose levels were monitored weekly using the Optium Xceed glucometer (Abbott, Bedford, USA).

### Echocardiography

After 8 weeks, echocardiography was performed as described previously [10]. Briefly, 30 rabbits were anesthetized by injection of 3% pelltobarbitalum natricum (30 mg/kg) into the marginal ear vein, and were placed on the table in the left lateral position. The thoracic wall was shaved, and six cardiac cycles of parasternal long-axis and short-axis images were recorded. The following 2D measurements were obtained from the parasternal long-axis view: left atrial anteroposterior diameter, interventricular septal thickness, left ventricular posterior wall thickness, left ventricular end-systolic dimensions and left ventricular end-diastolic dimensions. The left ventricular ejection fraction (LVEF) was also determined.

### Hemodynamic studies

Following echocardiographic examination, the rabbits were intravenously heparinized (1,200 IU) via the marginal ear vein. Carotid vein blood was collected for serum biochemical tests. The right carotid artery was isolated surgically. A catheter was inserted into the carotid artery to measure aortic systolic and diastolic blood pressure. Subsequently it was inserted into the left ventricle to measure the LV end-diastolic pressure.

### Histological studies

Following the hemodynamic studies, the hearts were removed. The wet weights (mg) of the left ventricle and the whole heart were measured. The left ventricular tissue was placed in 4% paraformaldehyde, embedded in paraffin, and cut into 5 mm cross-sections. The specimens were stained by hematoxylin and eosin (H&E) and cardiomyocyte cross-sectional area with the investigator blinded to the group of the rabbit. Ten random fields were studied per animal whereas the cardiomyocyte cross-sectional area was evaluated from myocytes cut in the short axis with an obvious nucleus. Masson's trichrome staining (Nanjing Jiancheng Bioengineering Institute, China) was used to evaluate ventricular interstitial fibrosis. Microphotographs were digitized using PHOTOSHOP 7.0 (Adobe, San Jose, CA) while cardiomyocyte cross-sectional area and areas of fibrosis were analyzed using Image PRO PLUS 7.0 SCION image software (Scion co., Frederick, MD, USA). The left ventricular interstitial collagen volume fraction (LVCVF) was defined as a quantitative ratio of the area of fibrosis to the area of the reference tissue.

### Serum biochemical and oxidative stress parameters, and ventricular myocardial NADPH oxidase activity

Serum biochemical parameters included: fasting insulin levels, blood urea nitrogen, creatinine, triglyceride, total cholesterol, high density lipoprotein and low density lipoprotein. Serum antioxidant enzyme activities, including nitric oxide, malondialdehyde, myeloperoxidase and superoxide dismutase, were measured using antioxidant enzyme activity kits (Nanjing Jiancheng Bioengineering Institute, China) according to the manufacturer's instructions. NOX activity was assessed using a GENMED kit (Genmed Scientifics Inc., Shanghai, China) by colorimetry. Proteins was extracted from the left ventricle and NOX activity was measured according to the manufacturer's instructions.

### Western blotting experiments

Frozen left ventricular tissues (100 mg) were grounded and lysed with RIPA lysis buffer. The lysates were boiled for 30 minutes and centrifuged at 12,000 rpm for 6 minutes. The proteins were loaded onto SDS-polyacrylamide gels and underwent electrophoresis. The proteins were then electro-blotted onto the PVDF membranes (Millipore, USA). Subsequently, the membranes were separately incubated with rabbit polyclonal to transforming growth factor-β1 (TGF-β1) antibody (Abcam, USA, 1:5000 dilution), nuclear factor kappa B (NF-κB) P65 antibody (Abcam, USA, 1:1000 dilution), p38 antibody (Abcam, USA, 1:1000 dilution), P-p38 antibody (Abcam, USA, 1:1000 dilution), MMP-9 antibody (Abcam, USA, 1:500 dilution), p22phox antibody(1:1000 dilution), gp91phox antibody(1:500 dilution), rac1 antibody(1:1000 dilution), and were incubated with appropriate peroxidase-conjugated secondary antibodies. Reactive blots were visualized by using Western Lightning^TM^ Chemiluminescence Reagent (Millipore) and quantified using densitometry.

### Statistical analysis

Statistical analysis was performed using SPSS 13.0 statistical software. All data are expressed as means ± standard deviation, Differences between groups were assessed using the Student's *t-test* (unpaired), and differences between three groups were analyzed by one-way ANOVA with post-hoc comparisons by the LSD test. A two-tailed *P-value* < 0.05 was considered statistically significant.

## References

[1] King H, Aubert RE, Herman WH (1998). Global burden of diabetes, 1995–2025: prevalence, numerical estimates, and projections. Diabetes care.

[2] Kayama Y, Raaz U, Jagger A, Adam M, Schellinger IN, Sakamoto M, Suzuki H, Toyama K, Spin JM, Tsao PS (2015). Diabetic Cardiovascular Disease Induced by Oxidative Stress. Int J Mol Sci.

[3] Rubler S, Dlugash J, Yuceoglu YZ, Kumral T, Branwood AW, Grishman A (1972). New type of cardiomyopathy associated with diabetic glomerulosclerosis. The American journal of cardiology.

[4] Trachanas K, Sideris S, Aggeli C, Poulidakis E, Gatzoulis K, Tousoulis D, Kallikazaros I (2014). Diabetic cardiomyopathy: from pathophysiology to treatment. Hellenic J Cardiol.

[5] Tse G, Lai TH, Yeo JM, Tse V, Wong SH (2016). Mechanisms of electrical activation and conduction in the gastrointestinal system: lessons from cardiac electrophysiology. Front Physiol.

[6] Tse G, Lai ET, Tse V, Yeo JM (2016). Molecular and electrophysiological mechanisms underlying cardiac arrhythmogenesis in diabetes mellitus. J Diabetes Res.

[7] Baynes JW, Thorpe SR (1999). Role of oxidative stress in diabetic complications: a new perspective on an old paradigm. Diabetes.

[8] Kowluru RA, Engerman RL, Kern TS (2000). Diabetes-induced metabolic abnormalities in myocardium: effect of antioxidant therapy. Free Radic Res.

[9] Uemura S, Matsushita H, Li W, Glassford AJ, Asagami T, Lee KH, Harrison DG, Tsao PS (2001). Diabetes mellitus enhances vascular matrix metalloproteinase activity: role of oxidative stress. Circ Res.

[10] Fu H, Liu C, Li J, Zhou C, Cheng L, Liu T, Li G (2013). Impaired atrial electromechanical function and atrial fibrillation promotion in alloxan-induced diabetic rabbits. Cardiol J.

[11] Liu T, Zhao H, Li J, Korantzopoulos P, Li G (2014). Rosiglitazone attenuates atrial structural remodeling and atrial fibrillation promotion in alloxan-induced diabetic rabbits. Cardiovasc Ther.

[12] Fu H, Li G, Liu C, Li J, Wang X, Cheng L, Liu T (2015). Probucol prevents atrial remodeling by inhibiting oxidative stress and TNF-alpha/NF-kappaB/TGF-beta signal transduction pathway in alloxan-induced diabetic rabbits. J Cardiovasc Electrophysiol.

[13] Qiu J, Zhao J, Li J, Liang X, Yang Y, Zhang Z, Zhang X, Fu H, Korantzopoulos P, Liu T, Li G (2016). NADPH oxidase inhibitor apocynin prevents atrial remodeling in alloxan-induced diabetic rabbits. Int J Cardiol.

[14] Seddon M, Looi YH, Shah AM (2007). Oxidative stress and redox signalling in cardiac hypertrophy and heart failure. Heart.

[15] Griendling KK, Sorescu D, Ushio-Fukai M (2000). NAD(P)H oxidase: role in cardiovascular biology and disease. Circ Res.

[16] Li JM, Gall NP, Grieve DJ, Chen M, Shah AM (2002). Activation of NADPH oxidase during progression of cardiac hypertrophy to failure. Hypertension.

[17] Stolk J, Hiltermann TJ, Dijkman JH, Verhoeven AJ (1994). Characteristics of the inhibition of NADPH oxidase activation in neutrophils by apocynin, a methoxy-substituted catechol. Am J Respir Cell Mol Biol.

[18] Qin F, Simeone M, Patel R (2007). Inhibition of NADPH oxidase reduces myocardial oxidative stress and apoptosis and improves cardiac function in heart failure after myocardial infarction. Free Radic Biol Med.

[19] Takahashi N, Kume O, Wakisaka O, Fukunaga N, Teshima Y, Hara M, Saikawa T (2012). Novel strategy to prevent atrial fibrosis and fibrillation. Circ J.

[20] Cai L, Kang YJ (2001). Oxidative stress and diabetic cardiomyopathy: a brief review. Cardiovasc Toxicol.

[21] Giacco F, Brownlee M (2010). Oxidative stress and diabetic complications. Circ Res.

[22] Baynes JW (1991). Role of oxidative stress in development of complications in diabetes. Diabetes.

[23] Williamson JR, Chang K, Frangos M, Hasan KS, Ido Y, Kawamura T, Nyengaard JR, van den Enden M, Kilo C, Tilton RG (1993). Hyperglycemic pseudohypoxia and diabetic complications. Diabetes.

[24] Shimizu M, Umeda K, Sugihara N, Yoshio H, Ino H, Takeda R, Okada Y, Nakanishi I (1993). Collagen remodelling in myocardia of patients with diabetes. J Clin Pathol.

[25] Sayed AA, Khalifa M, Abd el-Latif FF (2012). Fenugreek attenuation of diabetic nephropathy in alloxan-diabetic rats: attenuation of diabetic nephropathy in rats. J PhysiolBiochem.

[26] Inoguchi T, Sonta T, Tsubouchi H, Etoh T, Kakimoto M, Sonoda N, Sato N, Sekiguchi N, Kobayashi K, Sumimoto H, Utsumi H, Nawata H (2003). Protein kinase C-dependent increase in reactive oxygen species (ROS) production in vascular tissues of diabetes: role of vascular NAD(P)H oxidase. J Am Soc Nephrol.

[27] Balteau M, Tajeddine N, de Meester C, Ginion A, Des Rosiers C, Brady NR, Sommereyns C, Horman S, Vanoverschelde JL, Gailly P, Hue L, Bertrand L, Beauloye C (2011). NADPH oxidase activation by hyperglycaemia in cardiomyocytes is independent of glucose metabolism but requires SGLT1. Cardiovasc Res.

[28] Privratsky JR, Wold LE, Sowers JR, Quinn MT, Ren J (2003). AT1 blockade prevents glucose-induced cardiac dysfunction in ventricular myocytes: role of the AT1 receptor and NADPH oxidase. Hypertension.

[29] Zhang M, Kho AL, Anilkumar N, Chibber R, Pagano PJ, Shah AM, Cave AC (2006). Glycated proteins stimulate reactive oxygen species production in cardiac myocytes: involvement of Nox2 (gp91phox)-containing NADPH oxidase. Circulation.

[30] Maack C, Kartes T, Kilter H, Schafers HJ, Nickenig G, Bohm M, Laufs U (2003). Oxygen free radical release in human failing myocardium is associated with increased activity of rac1-GTPase and represents a target for statin treatment. Circulation.

[31] Price MO, Atkinson SJ, Knaus UG, Dinauer MC (2002). Rac activation induces NADPH oxidase activity in transgenic COSphox cells, and the level of superoxide production is exchange factor-dependent. J Biol Chem.

[32] Li J, Zhu H, Shen E, Wan L, Arnold JM, Peng T (2010). Deficiency of rac1 blocks NADPH oxidase activation, inhibits endoplasmic reticulum stress, and reduces myocardial remodeling in a mouse model of type 1 diabetes. Diabetes.

[33] Byrne JA, Grieve DJ, Bendall JK, Li JM, Gove C, Lambeth JD, Cave AC, Shah AM (2003). Contrasting roles of NADPH oxidase isoforms in pressure-overload versus angiotensin II-induced cardiac hypertrophy. Circ Res.

[34] Heymes C, Bendall JK, Ratajczak P, Cave AC, Samuel JL, Hasenfuss G, Shah AM (2003). Increased myocardial NADPH oxidase activity in human heart failure. J Am CollCardiol.

[35] Rameshwar P, Narayanan R, Qian J, Denny TN, Colon C, Gascon P (2000). NF-kappa B as a central mediator in the induction of TGF-beta in monocytes from patients with idiopathic myelofibrosis: an inflammatory response beyond the realm of homeostasis. J Immunol.

[36] Clerk A, Fuller SJ, Michael A, Sugden PH (1998). Stimulation of “stress-regulated” mitogen-activated protein kinases (stress-activated protein kinases/c-Jun N-terminal kinases and p38-mitogen-activated protein kinases) in perfused rat hearts by oxidative and other stresses. J Biol Chem.

[37] Sovari AA, Morita N, Karagueuzian HS (2008). Apocynin: a potent NADPH oxidase inhibitor for the management of atrial fibrillation. Redox Rep.

[38] Olukman M, Orhan CE, Celenk FG, Ulker S (2010). Apocynin restores endothelial dysfunction in streptozotocin diabetic rats through regulation of nitric oxide synthase and NADPH oxidase expressions. J Diabetes Complications.

[39] Tsai CT, Tseng CD, Hwang JJ, Wu CK, Yu CC, Wang YC, Chen WP, Lai LP, Chiang FT, Lin JL (2011). Tachycardia of atrial myocytes induces collagen expression in atrial fibroblasts through transforming growth factor beta1. Cardiovascular research.

